# Prosocial Preferences Condition Decision Effort and Ingroup Biased Generosity in Intergroup Decision-Making

**DOI:** 10.1038/s41598-020-64592-2

**Published:** 2020-06-23

**Authors:** Rima-Maria Rahal, Susann Fiedler, Carsten K. W. De Dreu

**Affiliations:** 1 Max Planck Institute for Research on Collective Goods, Bonn, Germany; 2 Leiden University, Leiden, Netherlands; 3 University of Amsterdam, Amsterdam, Netherlands

**Keywords:** Decision, Social behaviour, Visual system, Human behaviour

## Abstract

Ingroup favoritism and discrimination against outgroups are pervasive in social interactions. To uncover the cognitive processes underlying generosity towards in- and outgroup members, we employ eye-tracking in two pre registered studies. We replicate the well-established ingroup favoritism effect and uncover that ingroup compared to outgroup decision settings are characterized by systematic differences in information search effort (i.e., increased response times and number of fixations, more inspected information) and attention distribution. Surprisingly, these results showed a stronger dependency on the in- vs. out-group setting for more individualistic compared to prosocial participants: Whereas individualistic decision makers invested relatively less effort into information search when decisions involved out-group members, prosocial decision makers’ effort differed less between in- and outgroup decisions. Therein, choice and processing findings showed differences, indicating that inferences about the decision process from choices alone can be misleading. Implications for intergroup research and the regulation of intergroup conflict are discussed.

## Introduction

Decisions often affect not only the decision makers themselves, but also other people. Sometimes, these other people belong to the same group or community as the decision makers, and sometimes they belong to more or less rivaling outgroups. Research with experimental as well as natural groups has revealed that, in general, people are more generous when their decisions affect the outcomes of other individuals seen as part of one’s ingroup, rather than some rivaling outgroup^1^. At the same time, there is growing evidence that some people are more likely to display such ingroup bias than others: In intergroup contests, individuals with stronger prosocial preferences, who prefer fairness and care about own as well as other people’s welfare, are more likely to make costly contributions favoring their ingroup than individuals with weaker prosocial preferences^2–5^ (but see^6^).

Here we address the still poorly understood attentional mechanisms behind such moderated ingroup bias in cooperation and generosity. We do so using eye-tracking technology as a tool to capture the cognitive processes underlying human judgment and decision-making. Eye-tracking allows tracing overt attention allocation through recording gaze behavior during visual information search^7,8^, showing what is processed at a specific point in time, how much attention is allocated to specific pieces of information, and how much information is surveyed overall. Prior evidence shows that information about ingroup members is processed more thoroughly compared to information about outgroup members^9–12^. Going further, eye-tracking also harbours the potential to track fine-grained details of decision processes, such as the extent of information search (i.e., number of fixations) while decisions are formed, and the cognitive processing effort invested^11,12^. Moreover, the proportion of attention allocated towards or nonattendance of a specific piece of information as an indicator of the weight it is given during decision formation^13–17^ can be assessed. Accordingly, recording how information search unfolds over the course of the decision-making process in interdependent intergroup contexts can reveal the utility function underlying generous behavior towards the in- vs. outgroup.

When decisions affect both own and another individual’s outcomes, decision makers process the consequences of the decision alternatives for themselves as well as for others. Differences in generosity may thus be traced back to the extent to which individuals consider and weigh these consequences to both themselves and others^18,19^. A simple and straightforward model (other social preference models could also be considered^20–23^, but this paper uses a simple model as a starting point) represents utility *U* in terms of the trade-off between own and others’ payoffs as differences in the respective decision weights *w*_*own*_ and *w*_*other*_:

1 $${U}={{w}}_{{own}}\times {u}({\rm{o}}{\rm{w}}{\rm{n}}\,{\rm{p}}{\rm{a}}{\rm{y}}{\rm{o}}{\rm{f}}{\rm{f}})+{{w}}_{{other}}\times {u}({\rm{o}}{\rm{t}}{\rm{h}}{\rm{e}}{\rm{r}}{\rm{s}}\mbox{'}\,{\rm{p}}{\rm{a}}{\rm{y}}{\rm{o}}{\rm{f}}{\rm{f}})$$

From this equation, it follows that higher prosocial preferences (i.e., higher weighting of others’ payoffs) lead to an increase in utility for more generous decisions towards others (H1a). For instance, consider a decision maker with weak prosocial preferences (an individualist decision maker) with the utility function *U* = 0.9 × (own payoff) + 0.1 × (others’ payoff), and another decision maker with strong prosocial preferences (a prosocial decision maker) with the utility function *U* = 0.5 × (own payoff) + 0.5 ×(others’ payoff), as well as two choice options, Option A with the outcomes (7.50 [own], 5.20 [other]) and Option B with the outcomes (6.90 [own], 6.10 [other]). The prosocial decision maker would choose Option B (*U*_*A*_ = 6.35 < *U*_*B*_ = 6.5), and the individualist would choose Option A (*U*_*A*_ = 7.27 > *U*_*B*_ = 6.82). Now further assume that decision makers weight the welfare of ingroup members more than the welfare of outgroup members, which we model with a discounting parameter *β*:

2 $${U}={{w}}_{{o}{w}{n}\times {u}({\rm{o}}{\rm{w}}{\rm{n}}{\rm{p}}{\rm{a}}{\rm{y}}{\rm{o}}{\rm{f}}{\rm{f}})}+{\beta }\times {w}_{other}\times {u}({\rm{o}}{\rm{t}}{\rm{h}}{\rm{e}}{\rm{r}}{\rm{s}}\mbox{'}\,{\rm{p}}{\rm{a}}{\rm{y}}{\rm{o}}{\rm{f}}{\rm{f}}),\,{\rm{w}}{\rm{h}}{\rm{e}}{\rm{r}}{\rm{e}}:\,{\beta }=\{\begin{array}{cc} & 1,{\rm{i}}{\rm{n}}{\rm{g}}{\rm{r}}{\rm{o}}{\rm{u}}{\rm{p}}\\ 0 < {x} &  < 1,{\rm{outgroup}}\end{array}$$

In the above example, using a discounting factor *β* = 0.5 implies that a prosocial decision maker would now choose Option A (*U*_*A*_ = 5.05 > *U*_*B*_ = 4.975), and an individualist would still choose Option A (*U*_*A*_ = 7.01 > *U*_*B*_ = 6.515). It follows from Equation (2) first, that decision makers may be more generous to ingroup members than to outgroup members given the discounting factor *β* (H1b) and, second, that stronger prosocial preferences are associated with more ingroup bias in generosity, given prosocials’ larger weight placed on others’ outcomes (H1c).

The contribution of this paper is to address the cognitive processes underlying this well-established ingroup-biased generosity by investigating the processing level and attention to others’ outcomes as potential channels of the effect. Building on the attentional Drift Diffusion Model, utility differences do not only predict behavioral choices but also the cognitive effort spent on decision making. Note that processing effort is conceptualized here as the resources (i.e., time, attention) spent on making a choice, not as the (intuitive vs. deliberate) processing mode. According to drift diffusion models, information is accumulated in a stochastic process and integrated to identify the best option, i.e,. the option whose calculated Relative Decision Value exceeds a certain decision threshold. The attentional Drift Diffusion Model captures the speed of evidence accumulation over time in the drift rate parameter, which depends on utility differences between the choice options. When the differences in utilities are large, the evidence sampled is relatively more diagnostic for the Relative Decision Value, which therefore changes more drastically over time. In choice sets with large utility differences, the drift rate is therefore steeper compared to choice options with small differences in values^24,25^. Consequently, when utility differences are large and a strong preference for one choice option exists, less effort is required to make a decision than when utility differences are small^26^. Accordingly, effort should depend on social preferences and whether the partner belongs to the ingroup or the outgroup.

In the example above, when matched with an ingroup member, the individualist’s utility difference between the options is 0.45, while the prosocial’s utility difference is 0.15, given the respective weighting factors *w*_*own*_ and *w*_*othe*r_. When matched with an outgroup member, given the discounting factor *β* = 0.5, the individualist’s difference in utility between the options increases from 0.45 to 0.495, while the prosocial’s utility difference between the options decreases from 0.15 to 0.075. Therefore, when facing both in- and outgroup members, individualists process the decision problem less effortfully than prosocials^13^. Between the in- and outgroup setting, the individualist’s difference in utility changes by 0.045, while the prosocial’s utility difference between the options changes more drastically by 0.075. Therefore, there is a larger change in the utility difference between the in- and outgroup setting for the prosocial decision maker, which should lead to more pronounced differences in processing effort between the in- and outgroup setting than among individualistic decision makers in decision time (H2a), number of fixations (H2b), and amount of inspected information (H2c). Finally, because information that is weighted more highly in the decision process receives more attention while the choice is made^27,28^, Equation (2), in parallel to the expectations for choices and cognitive effort, implies that stronger prosocial preferences are associated with more visual attention directed at others’ outcomes^13^. Stronger prosocial preferences are expected to lead to ingroup bias in attention to others’ outcomes (H2d) compared to more individualistic preferences.

In sum, because the outgroup discounting factor has a bigger influence when preferences are prosocial rather than individualistic, we expect that the partner’s group membership should impact choices, attention and information processing effort more among people with strong rather than weak prosocial preferences. These predictions were examined in two experiments in which subjects were categorized into two groups, and made allocation decisions in decomposed dictator games with partners from their ingroup, and from the outgroup. Using eye-tracking technology, we assessed cognitive effort by considering the processing time required to make a choice, the number of fixations and the number of pieces of information being inspected. We additionally tracked the proportion of attention to outcomes to oneself and the partner among the decision alternatives. All hypotheses were preregistered on the Open Science Framework (OSF) prior to the start of the experiments (https://osf.io/wf5zy/ and https://osf.io/q8dfk/ all materials relating to methods and data are openly shared (https://osf.io/z2t2q/).

## Methods and Results for Study 1

In accordance with the preregistration, anticipating no-shows, we invited 110 participants, of which 84 took part in the study (55.95% women, *M*_*age*_ = 24.82, *SD*_*age*_ = 6.76). Due to the preregistered exclusion criteria (see below), the final sample consisted of 55 participants (80% power calculated to require *n* = 47, in our original power calculation using G*Power^29^, we assumed a medium effect size (*f*^2^ = 0.25), and *α* = 0.05, and omitted the multilevel structure of the dataset by estimating a linear regression because power analyses for multilevel models are not yet supported in this software.). Participants (mainly students) were recruited from the Decision*Lab* subject pool in Bonn, Germany, via the database system ORSEE^30^, and provided written informed consent prior to participation. The study (overview of the procedure in Figure S1) took about 40 minutes to complete, did not involve deception and was fully incentivized. Payoffs ranged from 5.30€ to 13.80€ (*M* = 9.52€, *SD* = 2.15€) and depended on participants’ decisions and performance as described below. This study received ethics approval by the Ethics Council of the Max Planck Society in the context of a generalized approval for experiments following the standardized protocol for experimental economics studies and was performed in accordance with these regulations.

In the first stage of the study, administered online via unipark (www.unipark.de), we assessed participants’ *prosocial preferences* with the Social Value Orientation (SVO) Slider Measure^31^. Participants encountered six monetary allocation problems, each asking them to choose between nine possible allocations of money between themselves and another person. For the payoff in this task, participants were randomly allocated to the role of the dictator or recipient and matched in pairs. After the study, one out of the six allocation problems was randomly drawn to be paid out. Depending on their role, participants were paid either in accordance with their own decision if they were a dictator, or if they were a receiver in accordance with the randomly selected decision of a matched dictator. From participants’ choices, we computed the SVO angle as a continuous measure of prosocial preference^26^. Note that earlier work often classified people into prosocials when 22.45° < SVO° < 57.15° and individualists when −12.04° < SVO° < 22.45°^31^.

Thereafter, to avoid choice-relevant stereotypes and beliefs about behavior often induced by real groups, we experimentally *manipulated group membership* by using an adaptation of the well-established Kandinsky-or-Klee procedure^32^. Five pairs of paintings consisting of one Kandinsky and one Klee clipping were shown^33^. Presentation order was randomized and participants were not informed about the painter. They indicated their liking of each painting presented (1 = dislike very much; 7 = like very much^34^. Based on their preferences, participants were then assigned to two groups: Giants (preference for Klee) or Titans (preference for Kandinsky), and informed of their group membership.

At least twelve hours after completing the online questionnaire, participants began the second stage of the study. They came to the laboratory where they were first introduced to a *group reinforcement task* that involved a competition with the outgroup. In a simplified version of the Serial Reaction Time Task^35^, participants were instructed to indicate the position of an asterisk shown randomly in one of four locations on the screen as quickly and accurately as possible. They were told that their reaction time would be added to their group’s total reaction time, and that every error they made would add five additional seconds to the group’s total, whereas finishing with less than five errors would reduce the group’s total by 50 seconds. At the end of each session, the running total of the groups’ reaction times in the group reinforcement task was compared. The group with the lower running total received a bonus payment of 3€.

**Figure 1:**
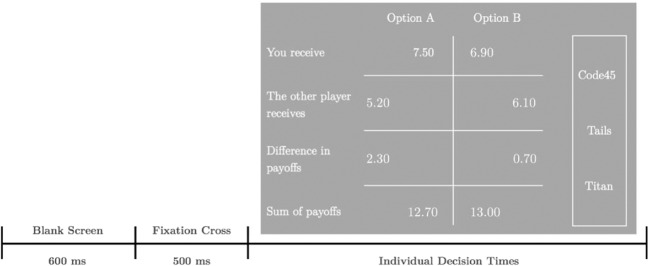
Money allocation task requiring a decision between two options for own and others’ payoff. Font sizes used in the experiment were smaller and spaces between items were larger than displayed here.

Hereafter, participants underwent the calibration procedures to set up the eye-tracking devices. To assess decision behavior, participants played 80 rounds of two-person *money allocation tasks* (minimal dictator games) and received information about the group membership of their respective partner for each trial. To manipulate the group setting, participants were assigned to one of two within-subject conditions: for 40 money allocations (28 target trials, 12 filler trials) their decisions concerned an ingroup member; and for another 40 money allocations their decisions concerned an outgroup member. The order of the trials was randomized. In each trial, participants decided between two options for allocating money between themselves and another participant. For each option, they were shown how this decision could affect their own payoff, the other person’s payoff, what the differences and sums of their own and the other person’s payoff would be (Figure 1). In each trial, participants could give up a certain amount of their own payoff that they would receive in the selfish option, to benefit the other player by choosing the prosocial option, where the prosocial option was randomly shown on the left or right side of the decision screen. In both options, the dictator would earn a higher payoff than the matched receiver. Unlike simple dictator games, in our design, the sums of payoffs differed between the options of the decomposed games to avoid any redundancies in the information about the two choice options. Redundancies would naturally result in lower gaze attendance due to uninformativeness and hence prohibit any clear interpretation of the attention distribution. To control for the resulting efficiency differences between the options and for the different cost-benefit ratio of the tasks, all choice analyses control for these variables. In the target trials, own payoffs varied between 2.40€ and 10€ (*M* = 7.18, *SD* = 2.04), others’ payoffs varied between 0.10€ and 7.80 € (*M* = 3.03, *SD* = 2.22). All participants were again assigned to either the dictator or recipient role and matched within the same pairs. Role assignment was determined by the random draw for their online decisions, in that participants that acted as a dictator (recipient) in the SVO task were now in the role of the recipient (dictator). One decision was again randomly selected per pair and determined the payoff for both players.

In each trial, information about the group membership of the matched receiver (Titan or Giant) was presented on the decision screen (Figure 1). Group membership information was displayed in a box together with two other, undiagnostic pieces of information about the other player: the outcome of a coin toss (heads or tails) and a personal code consisting of a random number they drew from an urn (e.g., “Code45”), in order to mirror natural decision contexts in which group membership information is rarely presented by itself, but rather jointly with other person-related information. Using additional undiagnostic information allowed us to additionally test whether attention towards group membership was specifically directed at the diagnostic information instead of just a by-product of simple curiosity during the experiment. The undiagnostic pieces of information hence function as negative controls. Analyzing differences in the proportion of attention spent on diagnostic vs. undiagnostic information about the other player showed that significantly more attention was directed to group membership (study 1: compared to outgroup decision settings are characterized by systematic differences in information = 58.16%, *SD*_group membership_ = 3.76%, *M*_coin_ = 20.06%, *SD*_coin_ = 2.94%, *M*_code_ = 21.79%, *SD*_code_ = 2.67%, study 2: *M*_group membership_ = 60.18%, *SD*_group membership_ = 3.12%, *M*_coin_ = 19.63%, *SD*_coin_ = 2.48%, *M*_code_ = 20.19%, *SD*_code_ = 2.20%. The location in which information about the matched player and the payoff relevant information (top, middle, bottom) was counter-balanced between subjects.

Eye movements were recorded during this task with the eye gaze binocular system (LC Technologies) with remote binocular sampling at a rate of 120 Hz and an accuracy of about 0.45°. Decision options were presented on monitors with a native resolution of 1280 × 1024 pixels and a refresh rate of 60 Hz (Samsung SyncMaster 740B (17 in, response time 8 ms), Samsung SyncMaster 931BF (18 in, response time 2 ms), Asus VB195T (18 in, response time 5 ms)).

At the end of the experiment, participants indicated (1 = strongly disagree, 7 = strongly agree) their identification with their in- and outgroups on eight items^36^ (“I identify with the group Giants [Titans]”, “I see myself as a member of the group Giants [Titans]”), and their explicit attitude towards their in- and outgroups on four items^34^ (“I like the group Giants [Titans]”, “The group Giants [Titans] is good”).

### Data Pre-processing

We defined three types of areas of interests (AOI) to assess fixations. AOIs containing payoff information or information on the identity of the matched person involved in the decision were defined as 100×100 pixels in size. AOIs containing labels describing the payoff information on the left side of the decision screens were contained by AOIs of 100×190 pixels. Fixations were identified with a 30 pixel tolerance in the summed deviation of points’ maximum and minimum coordinates on the x- and y-axes and a minimum duration of 50 ms^37^.

In line with the preregistration, the following data exclusions were made: Data from two participants had to be excluded because of missing gaze recordings. Additionally, one participant’s data was excluded for having indicated in a question included as a check for experienced experimenter demand that they decided against their personal preferences during the experiment. Further, data was excluded if participants failed to correctly indicate their outgroup, if who identified more with their outgroup than with their ingroup or liked the outgroup better than the ingroup (*N* = 8), to ensure that only participants who were successfully treated to consider themselves members of their ingroup more than their outgroup were included in the analyses. Additionally, as specified in the preregistration, trials in which participants allocated less than 50% of their fixations towards payoffs and identifying information (2.99%), as well as trials in which decision times were shorter than 200 ms and longer than 3 standard deviations above the overall mean decision time (1.91%) were excluded from further analysis to ensure high quality data. After these exclusions carried out according to the preregistration, 85.01% of all observations were retained. Additionally, a further exclusion criterion which had not been preregistered was used: For all process analyses, trials were excluded if participants failed to attend the group membership information, because they could not know about the relevant group context. To determine if participants attended to the group membership of the other player, conservatively, all gazes to the AOI containing this information were considered, regardless of the fixation duration. Overall, data from 55 participants was retained for further analyses. There was no evidence that the overall proportion of excluded trials depended on participants’ SVO angle (*β* = −0. 01, *t* = 1.01, *p* = 0.32).

### Generosity

**Figure 2:**
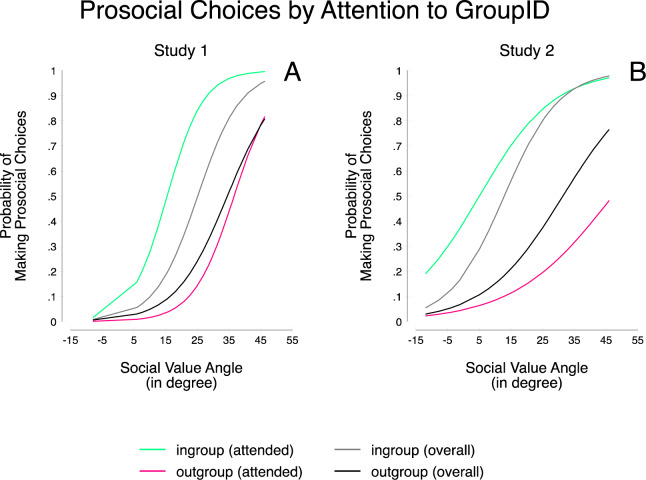
Probability of making prosocial choices in the money allocation task when facing in- or outgroup members depending on SVO. Separate for all trials, and trials in which group membership information was seen. Panel A: study 1; Panel B: study 2.

To analyze choice behavior, we employed a mixed effects repeated measures logistic regression using centered variables, predicting prosocial choices from SVO angle, group membership (ingroup vs. outgroup) and their interaction; we controlled for the percentage of monetary disadvantage incurred when making the prosocial choice and joint outcome (Table 1, Model 1). This revealed a main effect for SVO angle, indicating that participants with stronger prosocial preferences made more prosocial choices (H1a). Furthermore, we find that the odds of deciding prosocially increased by a factor of 27.07 when decisions involved an ingroup rather than outgroup partner (H1b). Finally, as predicted, we observed an interaction of SVO angle and group setting, showing that while individualists made similarly few prosocial choices in both group settings, we observed that more prosocial participants were more generous in the ingroup than in the outgroup setting (H1c, Figure 2A). The effect held when also including trials in which the receivers’ group membership was not attended to (Table 1, Model 2).

**Table 1:** Mixed effect repeated measures logistic regression predicting the odds ratio of making a prosocial choice in study 1 (Model (1): only for trials where participants attended to group membership information, Model (2): for all trials) and study 2 (Model (3): only for trials where participants attended to group membership information, Model (4): for all trials).

	Prosocial Decisions							
	Study 1				Study 2			
	(1) Group Attended		(2) All Trials		(3) Group Attended		(4) All Trials	
	*OR*	*z*	*OR*	*z*	*OR*	*z*	*OR*	*z*
SVO Angle	1.18***	5.45	1.14***	5.24	1.08***	3.97	1.10***	5.33
Group (0 = Out-, 1 = Ingroup)	27.07***	11.16	2.57***	8.32	21.91***	21.73	6.37***	19.58
SVO Angle x Group	1.02	1.15	1.03**	3.28	1.02***	2.49	1.03***	5.70
Percentage of Disadvantage	0.96**	−3.30	0.98***	−3.84	1.01	1.31	1.01	1.29
Efficiency	10.25***	4.02	9.57***	9.27	7.45***	7.23	10.20***	11.93
Constant	0.27**	−3.28	0.20***	−4.33	1.02	0.07	1.32	1.02
Observations	1006	4036	2086	4502				

*Note*. All predictors are centered. **p < *0*.01; ***p < *0*.001.

### Attention to Own Outcomes

**Figure 3:**
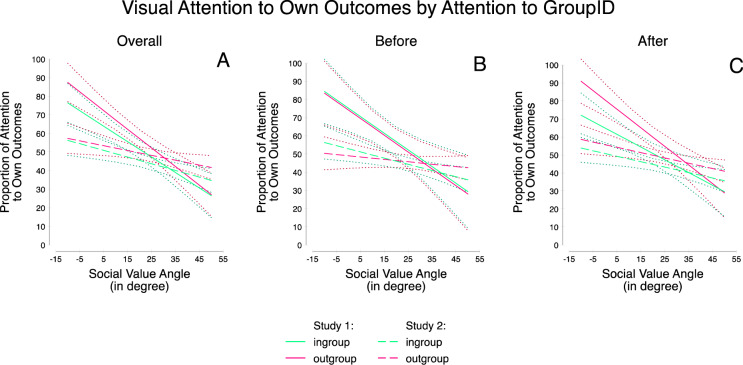
Proportion of attention to own outcomes depending on individual SVO, split by attention to the group identifier: overall (**A**), only before the group identifier was attended to (**B**) and only after the group identifier was attended to (**C**), with 95% confidence intervals for studies 1 and 2.

A mixed effects repeated measures linear regression, predicting the proportion of attention to own payoffs from SVO angle, group setting as well as the respective interaction (Table 2, Model 1), showed a significant main effect of SVO angle. Stronger prosocial preferences were associated with proportionally less attention to own payoffs. Moreover, participants proportionally attended less to their own payoffs when their partner was a member of the ingroup rather than the outgroup. Finally, an interaction effect between SVO angle and group setting was present, showing that the partner’s group membership influenced attention to own outcomes more among participants with weaker rather than stronger prosocial preferences (Figure 3A), in contrast to the original hypothesis (H2d).

**Table 2:** Mixed effects repeated measures linear regression predicting the proportion of attention to own outcomes from group setting and individual SVO in Studies 1 (Model 1) and 2 (Model 2).

	Attention to Own Outcomes			
	(1) Study 1		(2) Study 2	
	*β*	*z*	*β*	*z*
SVO Angle	−0.92***	−5.53	−0.31**	−2.89
Group (0 = Out-, 1 = Ingroup)	−6.24***	−5.59	−4.34***	−5.94
SVO Angle x Group	0.17*	2.27	−0.10*	−2.02
Trial	0.05*	2.16	0.05***	5.92
Constant	54.72***	22.32	43.45***	25.89
Observations	1006	2078		

*Note*. All predictors are centered. ∗*p* <0.05, ∗∗*p* <0.01, ∗∗∗*p* <0.001.

### Effort in Information Search

**Figure 4:**
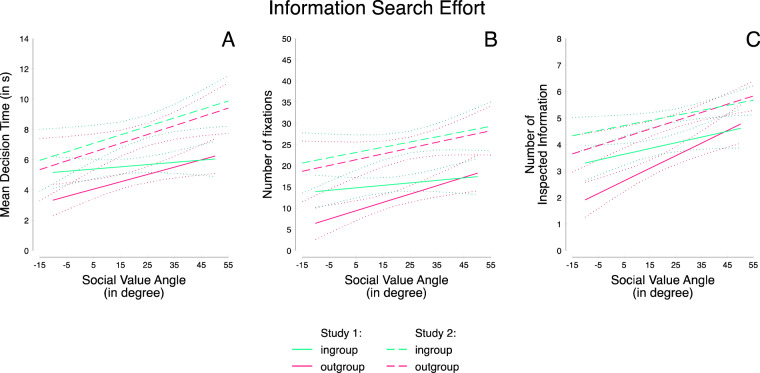
Decision effort (decision time (**A**), number of fixations (**B**) and number of inspected information (**C**) depending on individual SVO with 95% confidence intervals for studies 1 and 2.

We ran three mixed effects repeated measures linear regressions, predicting effort in information search operationalized via (i) decision times (H2a, Table 3, Model 1), (ii) number of fixations (H2b, Table 3, Model 3), and (iii) number of inspected information (H2c, Table 3, Model 5, for the correlation table across both studies, see Table S1). As predictors, SVO angle, group setting as well as the respective interaction were used. Results showed a significant main effect of SVO angle, indicating that stronger prosocial preferences were associated with more decision effort, i.e., a longer time to make decisions, more fixations and more information being inspected. A main effect of group setting showed that with ingroup rather than outgroup partners, effort increased as reflected in longer decision times, more fixations, and a higher number of inspected information. Finally, we found an interaction effect between SVO angle and group setting for all three effort measures. Results revealed that individuals with weak prosocial preferences invested less time, showed fewer fixations, and inspected less information when the partner was outgroup rather than ingroup. In contrast, individuals with stronger prosocial preferences showed reduced differences in effort between in- vs. outgroup settings (Figure 4). Thus, whereas behavioral generosity patterns were as predicted – stronger prosocial preferences were associated with greater generosity especially to ingroup members – the eye-tracking results showed an unexpected pattern. Before interpreting this set of results, we first examined its robustness in study 2.

**Table 3:** Mixed effects repeated measures linear regression predicting decision effort: log response times (Models 1 and 2), log number of fixations (Models 3 and 4), and proportion of inspected information (Models 5 and 6) from group setting and individual SVO in Studies 1 and 2.

	Log Response Time				Log Number of Fixations				Proportion of Inspected Information			
	(1) Study 1^a^		(2) Study 2^b^		(3) Study 1^a^		(4) Study 2^b^		(5) Study 1		(6) Study 2	
	*β*	*z*	*β*	*z*	*β*	*z*	*β*	*z*	*β*	*z*	*β*	*z*
SVO Angle	0.01**	2.72	0.01**	2.60	0.01**	2.59	0.01*	2.53	0.03*	2.32	0.03**	3.24
Group (0 = Out-, 1 = Ingroup)	0.21***	6.94	0.11***	5.23	0.26***	7.88	0.13***	5.91	0.44***	5.34	0.25***	4.26
SVO Angle x Group	−0.01***	−3.82	−0.01*	−2.18	−0.01***	−4.10	−0.01**	−3.25	−0.02**	−2.69	−0.01**	−3.20
Trial	−0.01***	−11.50	−0.01***	−20.68	−0.01***	−6.82	−0.01***	−17.65	−0.01***	−4.79	−0.01***	−16.56
Constant	1.74***	34.03	2.16***	46.13	2.75***	43.60	3.19***	59.13	4.76***	26.79	6.09***	45.16
Observations	1006		2078		1006		2078		1006		2078	

*Note*. Predictors are centered. ^+^*p* < 0.10, *p < 0.05; **p < 0.01; ***p <0.001. ^a^Results also hold for untransformed values. ^b^Assumption of normality was violated (Shapiro-Wilk test for response time showed *W* = 0.89, *z* = 12.43, *p* < 0.001 and for number of fixations *W* = 0.89, *z* = 12.41, *p* < 0.001. Results also hold for untransformed values, but do not reach standard levels of significance.

## Methods and Results for Study 2

In accordance with the preregistration we aimed at 90 complete data sets. Power calculations were based on study 1, where 95% power was estimated to be achieved with a sample size of *N* = 65 based on a medium effect size (*f*^2^ = 0.25), and *α* = 0.05, omitting the multilevel structure of the dataset. We additionally conducted a Monte Carlo simulations (with 500 replications) to estimate the appropriate sample size using the data from study 1 as our sampling frame^38^, utilizing the repeated measurement structure of our data. Specifically, we estimated a logistic repeated measurement model predicting altruistic choice by social preferences, intergroup context as well as their interaction controlling for the cost-benefit ratio and the difference in percentage of disadvantage present in each of the presented decisions. The analysis revealed that we would need only 20 participants to detect the two main effects but above 650 participants to detect the interaction effect with a power of 80%. Different seed values were used to ensure the stability of findings. The analysis script is available on the OSF (https://osf.io/pk49b/). Anticipating the usual no-show rate and calibration errors we invited 115 participants resulting in a total of 95 participants (68.13% women, *M*_*age*_ = 20.59, *SD*_*age*_ = 3.05, demographic information missing from four participants). Participants were recruited and compensated as before, with an additional flat payment given the longer duration of the sessions (total payoff between 6.00€ and 18.30€, *M* = 11.73€, *SD* = 2.74€). Experimental procedures and tasks were the same as for study 1 (overview of the procedure in Figure S1), except for the manipulation of group membership. Specifically, we adapted a procedure from Simon and Brown^39^ to increase group identification. Participants were shown 12 color boards (3 clearly blue, 3 clearly green, 6 an ambiguous mixture of blue and green) and asked to indicate their perception of each color board as blue or green. Based on their perceptions, participants were then assigned to two groups: Giants (more indications of blue) and Titans (more indications of green). To increase the relevance of the group and reinforce group membership, each participant also received a secret group password after being informed about their group membership. They were asked to memorize it so they could report it during the lab stage of the experiment. Providing the correct password would result in an additional payment of 1€. To increase group competition, they were also informed, that they would receive an additional “spying bonus” of 1€ if they could correctly name the outgroup’s password during the lab stage. In this event, all members of the outgroup present in their session would get no bonus even if they reported the correct password. They were instructed to keep their own group password secret to avoid spies from the other group costing them their bonus.

### Data Pre-Processing

AOI definition and data pre-processing for eye-tracking analyses were conducted as in study 1. One participant was excluded for deciding against her/his personal preferences during the experiment. Further, data was excluded from participants who failed to correctly indicate their outgroup, they identified more with their outgroup than with their ingroup, or liked the outgroup better than the ingroup (*N* = 10). Again, trials in which participants allocated less than 50% of their fixations towards payoff and identifying information (2.87%), as well as extremely short or long trials (2.59%) were excluded from the analyses. Overall, 85.19% of all observations and 77 participants were retained for further analyses. As in study 1, for process analyses all trials were excluded if participants failed to attend the group membership information. There was again no evidence that the overall proportion of excluded trials depended on participants’ SVO angle (*β* = − 0. 01, *t* = −0.06, *p* = 0.95).

### Generosity

As before we used a mixed effects repeated measures logistic regression predicting generosity from the SVO angle and the partner’s group membership (ingroup vs. outgroup), while controlling for the percentage of monetary disadvantage incurred when making the prosocial choice and the joint outcome (Table 1, Model 3; Figure 2B). As in study 1, results showed that stronger prosocial preferences were associated with greater generosity (H1a), and that the odds of being generous increased by a factor of 21.91 when participants had been matched with an ingroup instead of an outgroup member (H1b). Again, we found a significant interaction of SVO angle and group setting: While individuals with weaker prosocial preferences showed smaller differences in generosity between in- and outgroup settings, differences were larger for more prosocial participants (H1c). The effects held when also including trials in which the group membership information was not attended to (Table 1, Model 4).

### Attention to Own Outcomes

Regarding attention to own vs. others’ outcomes, we again ran a regression with SVO angle and the group setting as the independent variables, predicting the proportion of attention to own payoffs (Table 2, Model 2; Figure 3A). As in study 1, results showed significant main effects of SVO and the ingroup: Prosocials attended less to own outcomes than individualists, and when facing an ingroup member, the level of attention to own outcomes was lower than when facing an outgroup member. In line with our initial hypotheses (H2d), we found an interaction effect of SVO and group setting indicating a bigger difference in attention to own outcomes between in- and outgroup situations for more prosocial participants. This finding was in contrast to the result of study 1, where individualists had shown bigger differences in response to the group setting than prosocials. Analyzing these findings on a more fine-grained level, we restricted the analyses to the information search *after* the group identifier was first attended to (be it in a swift glance or a fixation), and found that the interaction effect in study 1 remained the same (Table S2, Model 1, and Figure 3C), while the interaction effect of study 2 did not present as significant (Table S2, Model 2, and Figure 3C). The main effects of SVO and group setting, as well as the interaction effects between these factors remained qualitatively the same and significant when rerunning the analyses on the dependent variables of decision effort for both Studies 1 and 2 (Table S3). From this it follows that the overall interaction effect of study 2 was most likely due to noise in the subsample *before* the group identifier was first attended to (Table S2, Model 4, and Figure 3B). Additionally, assessing the temporal dynamics of the attention distribution suggested fixation patterns were relatively stable over the course of the decision process (Figure S2).

### Effort in Information Search

Again, three regressions were conducted to assess effort in information search, predicting decision times (H2a, Table 3, Model 2), number of fixations (H2b, Table 3, Model 4), and number of inspected information (H2c, Table 3, Model 6) as a function of SVO angle, the group setting, and its interaction. Again, we found significant main effects of SVO and the group setting, as well as a significant SVO angle × group membership interaction. Whereas individuals with weaker prosocial preferences generally invested less effort when making a decision involving an outgroup rather than ingroup partner, participants with stronger prosocial preferences exerted similar amounts of effort when the partner was an in- or outgroup member (Figure 4).

## Conclusion and Discussion

Across two studies, we demonstrate behavioral ingroup-based generosity and illuminate the cognitive processes underlying such decisions, which are shown using eye-tracking. On the behavioral level, we found new evidence for stronger ingroup bias among decision makers with stronger rather than weaker prosocial preferences. Earlier work revealed such a pattern in intergroup contests in withingroup public goods, which we extend to ingroup biased generosity in a non-strategic setting. Thus, people with stronger prosocial preferences are more willing to reduce personal welfare to benefit another person from one’s in- rather than outgroup.

On the cognitive level, we demonstrate that decision effort and processing differed as a function of prosocial preferences and the group setting. First, stronger prosocial preferences were associated with more decision effort: longer time to make decisions, more fixations and more information being inspected (note that prosocial decisions were also associated with more effortful search processes (Table S4)). This finding fits predictions based on the attentional Drift Diffusion Model, and lends new support to the notion of relative utility weighting being reflected in the sampling process. The observation that stronger prosocial preferences are associated with more decision effort is at odds with work suggesting that cooperation is an intuitive choice made fast and without much deliberation^40,41^, and instead supports an account of increased effort invested in generosity relative to selfishness^42–47^. Investigating the influence of social preferences further, our analyses show that others’ outcomes were attended to more with increasing prosocial preferences. In line with the notion that information which is more important to the decision makers receives proportionally more visual attention, this finding supports the expectation that prosocial preferences affect the weight decision makers assign to others’ outcomes.

Our second, main finding is that processing effort differed systematically between in- and outgroup settings. Decision makers weighted others’ outcomes more, and invested more time and effort into acquiring information before making a decision when the partner was an in- rather than outgroup member. Possibly, humans are particularly concerned about the consequences of their decisions when ingroup members are involved and affected. The observed attentional neglect of the outgroup and the increased attention towards the ingroup fits other work showing that ingroup bias in cooperation and generosity is not primarily driven by a desire to hurt members of an outgroup, but rather by a desire to be good to one’s ingroup^1,2,48^. Results regarding whether individualistic persons show a stronger difference in their attention bias towards own payoffs between in- and outgroup situations in comparison to more prosocial participants did not replicate across the two studies, which we attributed to unanticipated noise in study 2. Therefore concluding only from study 1, the result that weak prosocial preferences bias attention more towards in- and outgroup individuals than strong pro-social preferences is in line with the pattern of results regarding our third main finding: the modulation of decision effort towards in- and outgroup members by prosocial preferences. In both cases, our expectations that people with stronger prosocial preferences would be more reactive to the in- vs. outgroup context were disconfirmed.

Behaviorally, stronger prosocial preferences were associated with larger differences in outgroup vs. ingroup generosity, and this difference was smaller among decision makers with more individualistic preferences. However, for attention to own outcomes and decision effort we observe the reverse pattern, in that individuals with stronger prosocial preferences presented with smaller processing differences between in- vs. outgroup situations than their individualistic counterparts. We did not anticipate the direction of this interaction pattern but are able to replicate it for the three dependent variables of decision effort across two studies. We suspect this pattern reflects a greater tension between individual-level preferences for selfishness and the group norm to be generous to the ingroup among individuals with weaker prosocial preferences compared to those with stronger prosocial preferences e.g.^49^. Less prosocial individuals may struggle more to balance their own interests and the group norm, while more prosocial individuals experience smaller conflict between these motives. Put differently, being partnered with someone from one’s ingroup creates a stronger choice dilemma with concomitant greater decision effort spent among individuals with weaker prosocial preferences. Future work varying the degree of conflict between individual preferences and group norms is needed to clarify this explanation further.

In sum, this work extends investigations of the underlying processes of ingroup favoritism to the domain of visual attention, opening multiple avenues for further research. In particular, it would be worthwhile to pursue different conceptualizations of social preferences, captured in different games modelling real-life choice behaviour. While this research has followed a simple model of social preferences^18^, other conceptualizations of decision makers’ regard for own and others’ outcomes, such as taking into account disadvantageous inequity^23^. Additionally, introducing strategic concerns by revealing the behavior and outcomes of matched players in previous interactions^21^ could prove valuable extensions for understanding cognitive processing involved in more complex decision scenarios. Allowing for real-time interactions between the decision makers and for feedback about own and others’ outcomes on a trial-by-trial basis further invites explorations of the influence of punishment motivations^16,46^ and reciprocity concerns^22^ on decisin processes in intergroup dilemmas. Moreover, studying decision processes at work when real groups are concerned could further inform the potential to derive interventions to decrease ingroup favoritism by directing attention and decision effort.

In conclusion, the present research takes a new look at intergroup research, moving beyond assessing mere choice behavior and towards considering the processes underlying these choices. First evidence sheds light on how much people with different social preferences care about others’ outcomes in the intergroup context, and how much they invest into searching for information when they make a decision that maximizes their own outcomes, that favors the ingroup or that supports the outgroup. With this glimpse at the cognitive processes underlying intergroup decisions, future avenues of research could investigate the implications of our findings for ameliorating ingroup favoritism, and intergroup conflicts.

## Supplementary information

Supplementary Material.
