# Supplementary material for: Prosocial Preferences Condition Decision Effort and Ingroup Biased Generosity in Intergroup Decision-Making
Source: Sci Rep. 2020 Jun 23;10:10132. doi: 10.1038/s41598-020-64592-2 (PMC7311554; doi:10.1038/s41598-020-64592-2)
Supplement: Supplementary file 1 — Supplementary Material. [file 41598_2020_64592_MOESM1_ESM.docx]

Supplementary Material:

Prosocial Preferences Condition Decision Effort and Ingroup Biased Generosity in Intergroup Decision-Making

Rima-Maria Rahal^1,2*^ Susann Fiedler^1^

Carsten K. W. De Dreu^2,3^

^1^ Max Planck Institute for Research on Collective Goods

^2^ Leiden University

^3^ University of Amsterdam

^*^ Correspondence should be addressed to:

Rima-Maria Rahal

Max Planck Institute for Research on Collective Goods

Kurt-Schumacher-Str. 10

53113 Bonn (Germany)

rahal@coll.mpg.de

Table S1

*Correlations of the measures of decision effort, pooled for Studies 1 and 2.*

|  | Decision  Time | Number of  Fixations | Number of  Inspected  Information | Proportion of Prosocial Decisions | Proportion of Attention to Own Outcomes |
| --- | --- | --- | --- | --- | --- |
|  | *r* | *r* | *r* | *r* | *r* |
| Decision Time | 1 |  |  |  |  |
| Number of Fixations | 0.92^***^ | 1 |  |  |  |
| Number of Inspected Information | 0.79^***^ | 0.81^***^ | 1 |  |  |
| Proportion of Prosocial Decisions | 0.41^***^ | 0.42^***^ | 0.54^***^ | 1 |  |
| Proportion of Attention to Own Outcomes | -0.53^***^ | -0.53^***^ | -0.66^***^ | -0.76^***^ | 1 |

*Note*. *∗∗∗ p < .*001, data is collapsed on subject level (*N*_total_ = 132) using mean values of the measures.

Table S2

*Mixed effects repeated measures linear regression predicting (1) proportion of attention to own outcomes, (2) log response times, (3) log number of fixations, and (4) proportion amount of inspected information from group setting and individual SVO in Study 2, before and after visually attending to group identifiying information for the first time.*

|  | Proportion of Attention to Own Outcomes | | | | | | | |
| --- | --- | --- | --- | --- | --- | --- | --- | --- |
|  | (1)  Study 1: before | | (2)  Study 1: after | | (3)  Study 2: before | | (4)  Study 2: after | |
|  | *𝛽* | *z* | *𝛽* | *z* | *𝛽* | *z* | *𝛽* | *z* |
| SVO Angle | -0.95^***^ | -4.74 | -0.87^***^ | -4.50 | -0.56^***^ | -4.37 | -0.30^**^ | -2.88 |
| Group (0 = Out-, 1 = Ingroup) | 0.48 | 0.85 | -8.89^***^ | -5.77 | -0.83 | -1.16 | -5.11^***^ | -6.07 |
| SVO Angle x Group | 0.03 | 0.89 | 0.33^**^ | 3.09 | -0.11^*^ | -2.23 | -0.01 | -0.10 |
| Trial | 0.16^***^ | 6.38 | 0.08 | 1.14 | 0.141^***^ | 8.92 | 0.13^***^ | 6.47 |
| Constant | 62.87^***^ | 21.51 | 53.14^***^ | 17.62 | 42.93^***^ | 21.37 | 40.86^***^ | 24.15 |
| Observations | 3661 | | 915 | | 4097 | | 2064 | |

*Note*. All predictors are centered. *∗ p < .*05, *∗∗ p < .*01, *∗∗∗ p < .*001.

Table S3

*Mixed effects repeated measures linear regression predicting decision effort: log response times (Models 1 and 2), log number of fixations (Models 3 and 4), and proportion amount of inspected information (Models 5 and 6) from group setting and individual SVO in Studies 1 and 2, restricted to the subsample of data after the group identifier was first gazed at.*

|  | Log Response Time | | | | Log Number of Fixations | | | | Proportion of Inspected Information | | | |
| --- | --- | --- | --- | --- | --- | --- | --- | --- | --- | --- | --- | --- |
|  | (1)  Study 1 | | (2)  Study 2 | | (3)  Study 1 | | (4)  Study 2 | | (5)  Study 1 | | (6)  Study 2 | |
|  | *𝛽* | *z* | *𝛽* | *z* | *𝛽* | *z* | *𝛽* | *z* | *𝛽* | *z* | *𝛽* | *z* |
| SVO Angle | 0.01^*^ | 2.23 | 0.01^**^ | 2.64 | 0.01^*^ | 2.27 | 0.01^*^ | 1.99 | 0.03^***^ | 3.39 | 0.03^**^ | 3.16 |
| Group (0 = Out-, 1 = Ingroup) | 0.20^***^ | 6.41 | 0.10^***^ | 5.00 | 0.32^***^ | 7.02 | 0.11^***^ | 3.54 | 0.60^***^ | 6.38 | 0.19^**^ | 2.83 |
| SVO Angle x Group | -0.01^***^ | -3.84 | -0.01^*^ | -2.25 | -0.01^***^ | -4.09 | -0.01^**^ | -2.69 | -0.03^***^ | -4.03 | -0.01^**^ | -2.70 |
| Trial | -0.01^***^ | -5.27 | -0.01^***^ | -21.98 | -0.01 | -0.75 | -0.01^***^ | -15.00 | -0.01 | -1.17 | -0.02^***^ | -16.42 |
| Constant | 1.69^***^ | 33.58 | 2.26^***^ | 48.16 | 2.10^***^ | 24.67 | 3.00^***^ | 51.33 | 3.77^***^ | 23.10 | 5.98^***^ | 45.60 |
| Observations | 915 | | 2064 | | 915 | | 2064 | | 915 | | 2064 | |

*Note*. All predictors are centered. *∗ p < .*05, *∗∗ p < .*01, *∗∗∗ p < .*001,

Table S4

*Mixed effects repeated measures linear regression with decision outcome as control, predicting decision effort: log response times (Models 1 and 2), log number of fixations (Models 3 and 4), and proportion amount of inspected information (Models 5 and 6) from group setting and individual SVO in Studies 1 and 2.*

|  | Log Response Time | | | | Log Number of Fixations | | | | Proportion of Inspected Information | | | |
| --- | --- | --- | --- | --- | --- | --- | --- | --- | --- | --- | --- | --- |
|  | (1)  Study 1 | | (2)  Study 2 | | (3)  Study 1 | | (4)  Study 2 | | (5)  Study 1 | | (6)  Study 2 | |
|  | *𝛽* | *z* | *𝛽* | *z* | *𝛽* | *z* | *𝛽* | *z* | *𝛽* | *z* | *𝛽* | *z* |
| SVO Angle | 0.01^+^ | 1.91 | 0.01^**^ | 2.58 | 0.01^+^ | 1.90 | 0.01^*^ | 2.42 | 0.02^+^ | 1.77 | 0.03^**^ | 3.08 |
| Group (0 = Out-, 1 = Ingroup) | 0.15^***^ | 4.62 | 0.09^***^ | 3.63 | 0.20^***^ | 5.41 | 0.10^***^ | 3.73 | 0.31^***^ | 3.32 | 0.15^*^ | 2.17 |
| SVO Angle x Group | -0.01^***^ | -4.40 | -0.01^*^ | -2.20 | -0.01^***^ | -4.69 | -0.01^**^ | -3.27 | -0.02^**^ | -3.22 | -0.01^**^ | -3.23 |
| Trial | -0.01^***^ | -11.52 | -0.01^***^ | -20.64 | -0.01^***^ | -6.80 | -0.01^***^ | -17.61 | -0.01^***^ | -4.76 | -0.01^***^ | -16.53 |
| Decision Outcome (0 = Selfish, 1 = Prosocial) | 0.15^***^ | 3.58 | 0.04 | 1.44 | 0.17 | 3.65 | 0.07^*^ | 2.31 | 0.37^**^ | 3.18 | 0.20^**^ | 2.64 |
| Constant | 1.68^***^ | 31.52 | 2.13^***^ | 45.98 | 2.68^***^ | 41.82 | 3.13^***^ | 58.28 | 4.62^***^ | 25.42 | 5.99^***^ | 43.56 |
| Observations | 1006 | | 2078 | | 1006 | | 2078 | | 1006 | | 2078 | |

*Note*. Predictors are centered. ^+^ *p* < .10, * *p < .*05; ** *p < .*01; *** *p < .*001.

Social Value Orientation Slides Measure

(Murphy, Ackermann, & Handgraaf, 2011)

*Study 1:* Klee vs. Kandinsky Procedure (Tajfel, Billig, Bundy, & Flament, 1971)

*Study 2:* Colour Boards (Simon & Brown, 1987)

*Study 1:* Reaction Time Task (Nissen & Bullemer, 1987)

*Study 2:* Reaction Time Task (Nissen & Bullemer, 1987) & Spying Bonus

Choices & Gaze Behavior

Identification (Doosje, Ellemers, & Spears, 1995)

Attitude (Pinter & Greenwald, 2010)

Correct declaration of group membership

SVO

Group Membership Manipulation

Group Reinforcement Task

Decision Task

Checks

Online Stage

Lab Stage

*Figure S1.* Overview of main aspects of the procedure in studies 1 and 2.

*Figure S2.* Attention distribution to the AOIs for own outcomes (self), other’s outcomes (other), the difference in payoffs (difference) and the sums of payoffs (sum) over the relative decision time elapsed, split by decision maker SVO and the group membership of the matched player, for Study 1 (Panels A through D) and Study 2 (Panels E through H).
